# Passive Transfer of A Germline-like Neutralizing Human Monoclonal Antibody Protects Transgenic Mice Against Lethal Middle East Respiratory Syndrome Coronavirus Infection

**DOI:** 10.1038/srep31629

**Published:** 2016-08-19

**Authors:** Anurodh Shankar Agrawal, Tianlei Ying, Xinrong Tao, Tania Garron, Abdullah Algaissi, Yanping Wang, Lili Wang, Bi-Hung Peng, Shibo Jiang, Dimiter S. Dimitrov, Chien-Te K. Tseng

**Affiliations:** 1 Department of Microbiology and Immunology, University of Texas Medical Branch, Galveston, TX 77555, USA; 2 Key Laboratory of Medical Molecular Virology of Ministries of Education and Health, School of Basic Medical Sciences, Fudan University, Shanghai 200032, China; 3 Department of Medical Laboratories Technology, College of Applied Medical Sciences, Jazan University, Jazan, Saudi Arabia; 4 Protein Interactions Section, Cancer and Inflammation Program, Center for Cancer Research, National Cancer Institute, National Institutes of Health, Frederick, MD 21702, USA; 5 Neuroscience & Cell Biology, University of Texas Medical Branch, Galveston, TX 77555, USA

## Abstract

Middle East Respiratory Syndrome coronavirus (MERS-CoV) has repeatedly caused outbreaks in the Arabian Peninsula. To date, no approved medical countermeasures (MCM) are available to combat MERS-CoV infections. Several neutralizing human monoclonal antibodies (mAbs), including m336, a germline-like human mAb, have been chosen as promising MCM for MERS-CoV. However, their clinical development has been hindered by the lack of a robust animal model that recapitulate the morbidity and mortality of human infections. We assessed the prophylactic and therapeutic efficacy of m336 by using well-characterized transgenic mice shown to be highly sensitive to MERS-CoV infection and disease. We found that mice treated with m336 prior to or post lethal MERS-CoV challenging were fully protected, compared to control mice which sufferered from profound weight loss and uniform death within days after infection. Taken together, these results support further development of m336 and other human monoclonal antibodies as potential therapeutics for MERS-CoV infection.

Middle East Respiratory Syndrome coronavirus (MERS-CoV), a recently identified novel coronavirus that causes fatal acute respiratory illness in human, was initially isolated from a Saudi Arabian patient with acute pneumonia and renal failure in June 2012^1^. As of July 6th, 2016, 1,782 cases with 634 deaths have been confirmed in 27 countries (http://www.who.int/emergencies/mers-cov/en/). While the clinical presentations of MERS-CoV are very similar to those of SARS-CoV, phylogenetic analysis has revealed that MERS-CoV is genetically more closely related to bat coronaviruses than to SARS-CoV, suggesting that it may have originated from bats before evolving into a human pathogen^2^,^3^,^4^,^5^,^6^,^7^,^8^. Of note, MERS-CoV has been detected in dromedary camels, and a high prevalence of MERS-CoV-specific antibodies can be found in camels from some regions in the Middle East and Africa^9^,^10^,^11^,^12^,^13^,^14^. A recent study revealed the co-circulation of several human coronavirus species and MERS-CoV lineages in dromedary camels in Saudi Arabia, including a recombinant strain, which has been a dominant isolate from patients since December 2014 and subsequently led to human outbreaks in 2015^15^. This study suggested that the dromedary camel may serve as an important reservoir and that MERS-CoV may represent a continuous and long-term threat to people, particularly those who interact closely with camels in the Arabian Peninsula. Even though MERS-CoV presently has limited human-to-human transmission^2^,^16^, the high mortality rate of this virus and limited information on the mechanism able to confer increased human-to-human transmission have raised concerns of a potential MERS pandemic. Indeed, the recent outbreaks in Korea and the appearance of super-spreading events indicate that MERS-CoV has the ability to cause large outbreaks outside of the Arabian Peninsula^17^,^18^,^19^. Currently, no approved vaccines or drugs are available to treat this viral infection. These facts highlight an urgent need to develop potent prophylactic and therapeutic agents to fight this lethal virus.

Similar to other coronaviruses, MERS-CoV uses the envelope spike (S) glycoprotein, a class I transmembrane protein, for interaction with its cellular receptor for binding, fusion and entry into the target cell^20^. The receptor binding domain (RBD) located in the S1 domain of the MERS-CoV spike is responsible for binding to the well-characterized cellular receptor identified as DPP4 (CD26) and is, therefore, critical for binding and entry of the virus^20^,^21^,^22^. Therefore, neutralizing antibodies capable of blocking such interaction could be promising preventive and/or therapeutic candidates. Recently, human monoclonal antibodies (mAbs) capable of neutralizing MERS-CoV have been identified and characterized by several research groups^23^,^24^,^25^,^26^,^27^,^28^. These antibodies have been isolated from naive human antibody libraries, from transgenic “humanized” mice, or from B cells of an infected individual, and they recognize different epitopes on MERS-CoV RBD. One of the most potent mAbs, m336, is a germline-like antibody identified from a very large (~10^11^ size) phage-displayed antibody library derived from B cells of healthy donors. This mAb exhibits exceptionally potent neutralizing activity (IC_50_ = 0.005 μg/ml) *in vitro*^23^. Moreover, because its epitope almost completely (~90%) overlaps with the receptor-binding site of DPP4 on MERS-CoV RBD, as is evident by its recently solved crystal structure^29^, the probability of generation of resistant mutants may be absent or very low. Notably, although the functions of these mAbs have been extensively characterized *in vitro*, their further clinical development has been hindered by the lack of an effective animal model of MERS-CoV infection. MERS-CoV cannot infect small laboratory animals (e.g., mice, hamsters and ferrets) as a consequence of species-specific differences in DPP4, while only causing mild-to-moderate symptoms in rhesus macaques. Marmosets, which are more susceptible to MERS-CoV, developed a moderate-to-severe disease, but limited availability and high cost have hampered their use^30^. Rabbits can be infected, but the infectious virus is challenging to detect^31^,^32^. It was found that the expression of human DPP4 could overcome the lack of susceptibility in normal mice. With prior transduction of adenoviral human DPP4-expressing vectors, mice became susceptible to MERS-CoV infection without revealing any measurable clinical manifestations^33^. In contrast, transgenic (Tg) mice with the human DPP4 gene integrated into the genome readily developed acute morbidity (weight loss), and uniform death occurred within a week^34^,^35^, making it an ideal preclinical model for the development of vaccines and treatments against MERS.

Some of the aforementioned human neutralizing monoclonal antibodies have been shown to protect engineered human DPP4-expressing mice and the naturally permissive rabbits, entirely based on their ability to inhibit MERS-CoV infection and/or alleviate histopathology of the lungs^27^,^28^,^36^,^37^. To further verify the protective efficacy of these human monoclonal antibodies, particularly m336, against MERS-CoV infection, it is highly desirable to use the well-characterized human DPP4 Tg mice known to result in acute disease (weight loss) and death^34^,^35^.

By using this highly permissive Tg mouse model, we evaluated the prophylactic and therapeutic efficacy of m336 mAb *in vivo*. We report in this study for the first time that treatment of Tg mice with a single-dose of m336 antibody prior to or after challenging with 1,000 LD_50_ of MERS-CoV protected mice from the lethality in a dose-dependent manner, thereby representing the first antibody tested for its protective efficacy against lethal MERS-CoV infection.

## Results

### Prophylactic efficacy of MERS-CoV RBD-specific human monoclonal antibody, m336

**Figure 1:**
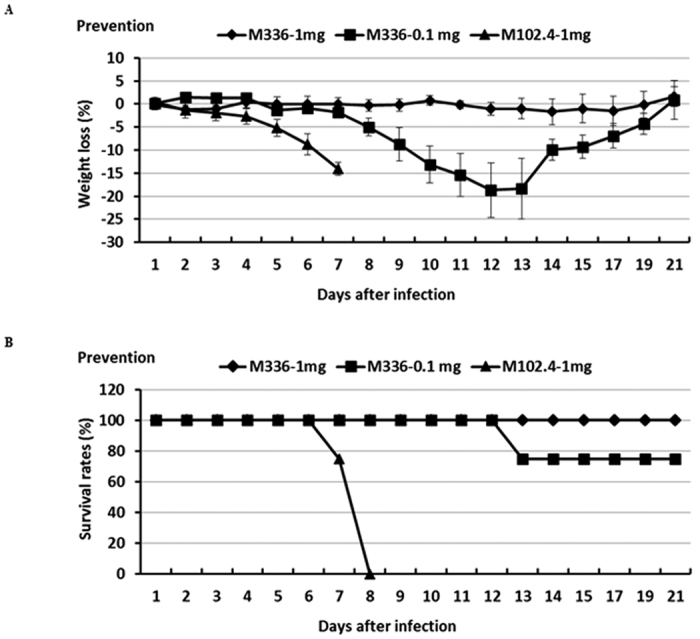
Prophylactic efficacy of mAb m336 in protecting Tg mice against lethal dose MERS-CoV challenge. Tg mice were treated (i.p.) with m336 antibody 12 h before challenge (i.n.) with 10^4^ TCID_50_ of MERS-CoV. An irrelevant human mAb, m102.4, was included as the control. Challenged mice were monitored daily for the weight loss **(A)** and accumulated mortality **(B)**, expressed as percent (%) weight loss and survival, respectively.

We established a Tg mouse model which is profoundly sensitive and susceptible to MERS-CoV infection, as determined by high viral titers in the lungs, as well as a high rate of morbidity and mortality^34^,^38^. Equipped with this small animal model of human MERS-CoV, we investigated the protective efficacy of mAb m336. To accomplish this, each group (n = 6) of mice was treated via the intraperitoneal (i.p.) route with two different doses: 0.1 mg and 1 mg per mouse diluted in 100 μl PBS, and challenged intranasally (i.n.) at 12 h post treatment with 10^4^ TCID_50_ (i.e., 1,000 LD_50_) of MERS-CoV in a volume of 60 μl^38^. Challenged mice were monitored daily for clinical manifestations (weight loss)  and mortality. As shown in Fig. 1, the group treated with 1 mg mAb survived viral infection without showing any clinical symptoms. These mice initially showed either no weight loss or recovered from mild weight loss within three days (Fig. 1A). On the other hand, the group treated with 0.1 mg mAb showed a gradual weight loss (15–20%) until day 13 just before starting to recover (Fig. 1A). All surviving mice (one died on day 13 in mice treated with 0.1 mg of m336) continued to recover and appeared well up to 21 dpi when the experiment was terminated (Fig. 1B). All MERS-CoV-challenged mice pretreated with a high dose (1 mg) of irrelevant mAb m102.4 exhibited profound weight loss (>15%) and succumbed to infection with 100% mortality by day 8 p.i. (Fig. 1A,B).

### Therapeutic efficacy of MERS-CoV RBD-specific human monoclonal antibody, m336

**Figure 2:**
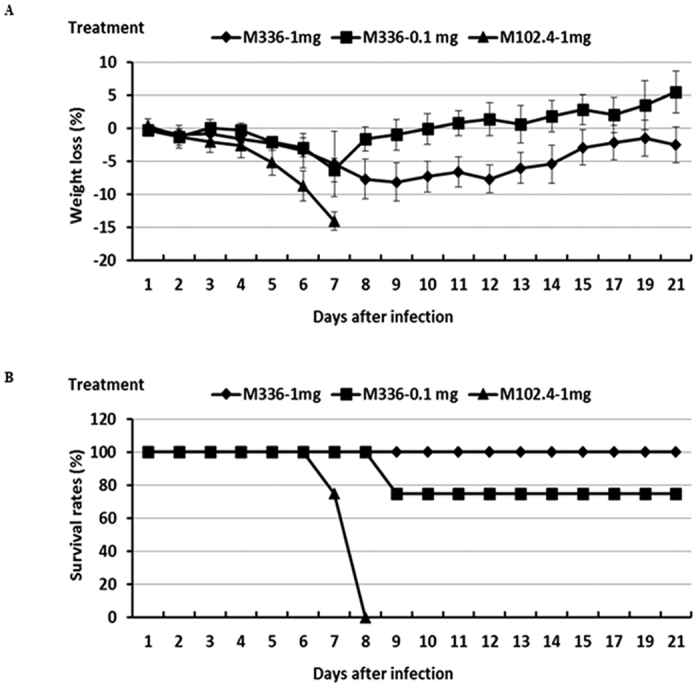
Therapeutic efficacy of mAbm336 in protecting Tg mice against lethal MERS-CoV challenge. Tg mice were treated (i.p.) with human m336 antibody 12 h after infection (i.n) with 10^4^ TCID_50_ of MERS-CoV. An irrelevant mAb, m102.4, was also included as the control. Challenged mice were monitored daily for the weight loss **(A)** and accumulated mortality **(B)**, expressed as percent (%) weight loss and survival, respectively.

To determine the therapeutic potential of this human monoclonal m336 antibody, groups of mice (N = 6 per group) were challenged (i.n.) with 10^4^ TCID_50_ of MERS-CoV (i.e., 1,000 LD_50_) in a volume of 60 μl and then treated (i.p.) 12 hours later with a single dose of either 1 mg or 0.1 mg of m336 or 1 mg of m102.4 antibody (control) in 100 μl per mouse, followed by monitoring daily for wellbeing (weight loss and other clinical manifestations) and mortality of mice. We noted that whereas treatment with 1 mg of m336 antibodies was effective in the protection against the lethality caused by MERS-CoV infection, it failed to protect mice fully from the onset of clinical illness (weight loss). Specifically, all of the challenged mice treated with 1 mg of m336 antibody suffered an attenuated (<10%), and transient weight loss until day 9, and gradually recovered to day 21 when the experiment was terminated (Fig. 2). Similarly, challenged mice treated with a low dose of 0.1 mg of m336 antibodies suffered from attenuated and transient weight loss until day 7 p.i. and gradually recovered. However, we noted a single death at day 9 in this low dose treatment group (Fig. 2). As expected, all mice treated with a single dose of 1 mg of control m102.4 antibody exhibited profound weight loss (>15%) and succumbed to MERS-CoV infection with 100% mortality by day 8 p.i. (Fig. 2). Taken together, these results indicate that this MERS-CoV RBD-specific human m336 antibody can be highly effective as prophylactic or therapeutic modalities in protecting highly permissive transgenic mice against MERS-CoV infection and disease.

### Lung virus titers in mice treated with MERS-CoV RBD-specific human monoclonal antibody, m336

**Figure 3:**
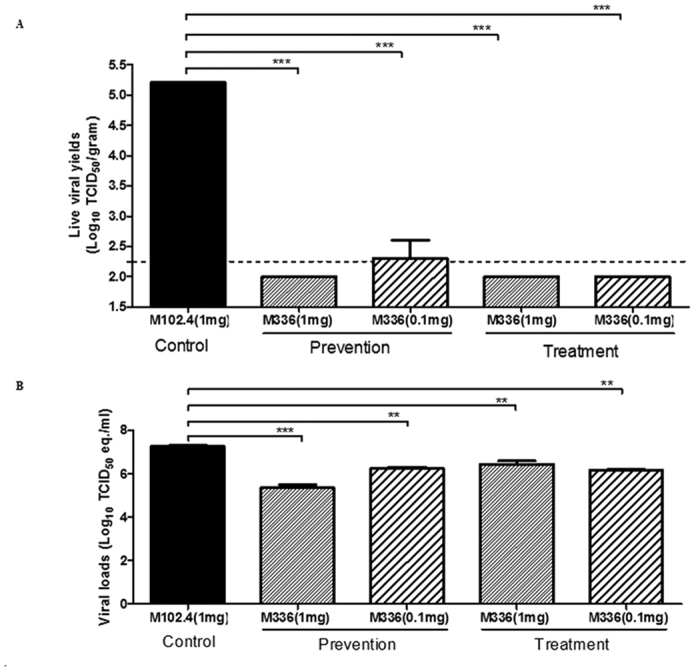
Treatment with m336 antibody significantly inhibited MERS-CoV infection within the lungs. Lung specimens collected at day 2 after viral challenge were processed for assessing the viral titers by using both Vero E6-based infectivity assay and qRT-PCR targeting upstream E gene of MERS-CoV, and expressed as log_10_ TCID_50_/gram and log_10_ TCID_50_ equivalent (eq.)/gram, respectively. **(A)** Prophylactic and therapeutic efficacy of human m336 antibody treatment in reducing the lung titers of infectious virus. **(B)** Prophylactic and therapeutic efficacy of human m336 antibody in reducing the titers of viral RNA. The data shown are representative of at least two independently conducted assays using the same samples. Data is presented as Mean ± standard error (SE). ***P < 0.001 as determined by using Student’s t test.

We also investigated the protective mechanism of m336 against MERS-CoV by determining the lung virus titers in challenged mice at day 2 after treatment. Specifically, we sacrificed two mice (out of 6) in each group, as described above for Figs 1 and 2 and their lung specimens were harvested for determining viral titers by using via Vero E6 cell-based infectivity assay and quantitative PCR (Q-PCR)-based assay targeting the upstream E gene of MERS-CoV. As shown in Fig. 3A we were unable to recover infectious virus from any mouse treated with 1 mg of m336 antibody either before or after challenge with MERS-CoV. However, we were able to detect a barely detectable infectious virus, with the limit of detection (LOD) of 2.3 log TICD_50_/g, from a single mouse receiving 0.1 mg of m336 prior to viral challenge. These results indicated that mAb m336 most likely confers protection from lethal challenge by restricting viral replication within the lungs, thereby preventing viral infection in the brains and other organs.

Titers of viral RNA copy number, as shown by qRT-PCR assays, were also compared among groups having different doses of mAbs. Lungs of infected mice were harvested on day 2 post- and pre-virus challenge group. All groups exhibited detectable viral RNA. Titers were significantly lower than those in the control group in all m336-treated groups. In the pretreatment group, mice treated with 1 mg of m336 showed a 2-log reduction in viral RNA detection, while a ~1 log reduction in viral numbers was seen in mice treated within 0.1 mg m336 when compared to mice receiving control mAb m102.4. In the post-treatment group, a smaller (~1 log) difference in viral RNA copy number (compared to that in the pretreatment group) was observed between mice treated with 1 mg antibody compared with those receiving control antibody, while a more than 1 log reduction in viral RNA number was seen in mice treated with 0.1 mg m336 when compared to mice receiving control mAb (Fig. 3B). These data indicate that m336 confers significant protection to mice when administered pre- or post-viral challenge. Taken together, these results suggest to us that the epitope targeted by this exceptionally potent RBD-specific m336 antibody has a great potential for further development as a potent preventive and therapeutic agent in the future.

### Treatment with m336 attenuates lung pathology associated with MERS-CoV infection

**Figure 4:**
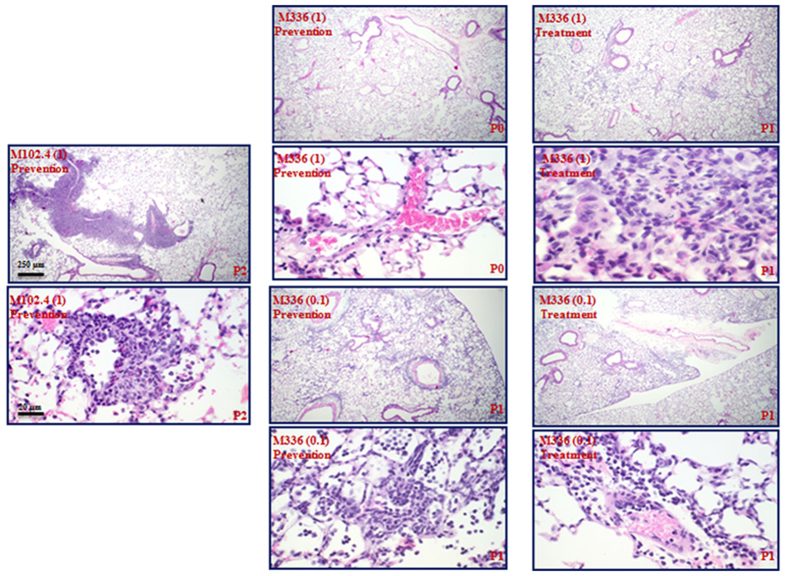
Treatment with human m336 antibody significantly attenuates lung pathology of mice challenged with MERS-CoV. Lung specimens collected at day 2 post infection were fixed, appropriately processed, and H&E-stained for assessing the lung pathology. The lung pathology scores were graded from 0–3 (none, mild, moderate, and severe), based on the extent of mononuclear cell infiltration. Left: mice treated with 1 mg m102.4 control antibody 12 h prior to viral infection. Middle: mice treated with 1 or 0.1 mg of m336 antibody 12 h prior to viral infection as indicated. Right: mice treated with 1 or 0.1 mg of m336 antibody 12 h after viral infection as indicated.

The effect of m336 antibody treatment on the pulmonary pathology associated with MERS-CoV infection was evaluated by using formalin-fixed, paraffin embedded, and hematoxylin/eosin (H&E)-stained lung specimens harvested at day 2 p.i. Pulmonary pathology was noted in all mice that were treated with different doses of m336 or control m102.4 antibodies either before or after viral infection. On a severity scale of 0 to 3 (none, mild, moderate, severe), H&E-stained samples from mice pretreated with 1 mg and 0.1 mg of m336 antibody were graded 0 and 1, respectively, for perivascular and intra-alveolar infiltration of mononuclear cells, including lymphocytes, macrophages/monocytes (Fig. 4, Middle panel), whereas those obtained from mice that received post-infection treatment with either dose of m336 were graded 1 (Fig. 4, right panel), compared to the grade 2 assigned to mice received control antibody treatment prior to infection (Fig. 4, left panel).

## Discussion

MERS-CoV has attracted significant basic research and clinical studies since it was first discovered in early 2012. Even though the transmissibility of MERS-CoV among humans remains low at present, as a mutation-prone RNA virus, it could eventually evolve into a highly communicable and more virulent human pathogens. This emphasizes the urgent need for the development of an effective antiviral therapy which could restrict the spread of this deadly disease. In other viral infections, neutralizing antibodies have been shown to protect the host from disease progression and/or reduce the severity of clinical symptoms. Passive immunotherapy for prophylaxis and treatment of infectious viral diseases has been widely used for many decades^39^,^40^,^41^,^42^,^43^. Passive transfer of neutralizing antibodies is also a promising strategy for both prophylaxis and treatment against MERS-CoV infection. To this end, we and others have successfully demonstrated the protective efficacy of specific human neutralizing monoclonal antibodies in animal models of MERS-CoV infection^23^,^24^,^26^,^28^. Among a panel of MERS-CoV-specific mAbs generated by using a vast phage display library^23^, we identified three mAbs which specifically bind to the MERS-CoV RBD with very high affinity. Among these three identified, we noted that mAb m336 exhibited the highest potency in neutralizing live MERS-CoV. Here, we further characterized this novel human mAb in our Tg mouse model of MERS-CoV infection and showed prophylactic and therapeutic protection of mice treated with m336 before and after a lethal challenge with the virus, respectively. Thus, mAb m336 is highly promising as a potent inhibitor for urgent prophylaxis in adjunctive treatment for patients infected with MERS-CoV.

In our studies, we noted that passively transferred with 1 mg and 0.1 mg of m336 monoclonal antibodies to individual mice 12 h prior to challenge with 1,000 LD_50_ of MERS-CoV resulted in 100% and 75% protection against lethality, respectively (Fig. 1), suggesting that using 0.1 mg m336/mouse as a prophylaxis is suboptimal to completely neutralize viral infection, thereby allowing residual viruses to replicate within lungs during the course of infection. These data demonstrate that m336 confers a dose-dependent reduction of MERS-CoV infection, corroborating lower viral RNA levels and live virus isolation determined for these mice when compared to control mice. Our study also confirmed the therapeutic efficacy of m336 in a dose-dependent manner. Similar to the prophylactic studies, administration of a single-dose of m336 antibody at a concentration of either 1 or 0.1 mg per mouse at 12 h after MERS-CoV challenge provided 100% and 75% protection, respectively, against infection-induced lethality, accompanied by reduced viral loads (both infectious virus and viral RNA) within the lungs. However, we also noted the recovery of bodyweight loss and the reduction of viral loads in mice treated with 1 mg of m336 at 12 hrs after infection were slower than those treated with 0.1 mg of m336, as shown in Figs 2A and 3B, respectively. While there is no clear evidence showing an adverse impact on the overall wellbeing of mice imposed upon treatment with 1 mg of m336 antibody before MERS-CoV challenge (Fig. 1), it is difficult to completely rule out the existence of subtle “yet-to-be investigated” high-dose drug toxicity. We speculate that such a subtle high-dose drug toxicity in the phase of acute and dynamic MERS-CoV infection initiated at 12 hrs before treatment with 1 mg of m336 could exacerbate drug toxicity, resulting in reduction of appetite and antiviral capacity. However, such a negative impact imposed upon high-dose treatment of virally infected mice appeared to be transient and did not irreversibly alter the final outcome of infection, as judged by the mortality (Fig. 2B). Additional studies, especially the pharmacokinetics and the dosing frequency of m336 are warranted in the future to optimize preventive and therapeutic strategies with this promising antibody.

The transgenic mice that we used for evaluating the prophylactic and, especially, the therapeutic efficacy of this m336 antibody are extremely sensitive to MERS-CoV infection and disease, with LD_50_ and ID_50_ of 4.5 and 0.4 TCID_50_ of MERS-CoV, respectively (data not shown), titers which are lower than our original estimations^38^. Such a striking ability of this m336 antibody, as a prophylactic or therapeutic agent, to significantly protect these transgenic mice against challenge with 1000 LD_50_ of MERS-CoV is highly impressive. The RBD of the MERS-CoV, targeted by this m336 antibody, is highly conserved among various clinical isolates and the mutation rate of this RBD appears to be extremely low, compared to that of other RNA viruses^23^,^28^, thereby making the development of escape mutants to m336 unlikely. However, a combination treatment with multiple neutralizing mAbs targeted at different epitopes or the MERS-CoV-specific HR2P fusion inhibitor targeting the HR1 domain of the S2 subunit of the MERS-CoV S protein^38^,^44^ could be desirable.

By immunizing mice with RBD of MERS-CoV S protein, Li, Y. *et al*. recently developed a humanized mAb, named 4C2h, that exhibited strong neutralizing activity with ND_50_ of ~0.71 and ~6.25 μg/ml against the pseudotyped and live MERS-CoV, respectively^36^, which are about 100-fold less potent than m336 (ND_50_ = 0.005 and 0.07 μg/ml against the pseudotyped and live MERS-CoV, respectively)^23^. Using Ad5-hCD26-transduced mouse model^33^, they demonstrated that intravenous administration of a single dose of 4C2h one day before or after the MERS-CoV challenge resulted in reduction of viral titer by 2 log at 3 dpi. However, intraperitoneal administration of m336 to our hDPP4 Tg mice lead to the reduction of viral titer as high as 4 log at 2 dpi. Since MERS-CoV challenged Ad5-hCD26-transduced mice showed no severe disease, the effect of 4C2h on the weight loss and mortality in these mice is unavailable. Additionally, unlike hDPP4 transgenic mice that we used in this study with well-defined hDPP4 expression as well as 50% lethal dose (LD_50_) and infectious dose (ID_50_), the intensities of hCD26 expression among the Ad5-hCD26-transduecd mice are variable, ranging from undetectable to a high level^45^. Although both 4C2h and m336 bind to the RBD of MERS-CoV S protein, some of the critical amino acid residues recognized by these two mAbs are different^29^,^36^. The epitope of m336 overlaps extensively with the DPP4-binding site, which is composed of MERS-CoV RBD residues N501-K502, S504, F506, D510, E513, W535-R542, W553, V555, S557 and S559^29^. The epitope of mAb 4C2, the parental mouse mAb of 4C2h, only overlaps with partial of DPP4-binding site, which is composed of five RBD residues, W535-E536 and D539-R542. Most of other RBD amino acids recognized by 4C2, including Y397-N398, K400, L495-K496, P525 and V527-S532, are not located on the DPP4-binding site, indicating that the neutralization efficacy of 4C2h is largely attributed to the steric hindrance created by its binding with MERS-CoV RBD^36^.These results suggest that combinational use of 4C2h and m336 may exhibit synergistic antiviral effect against both wild-type strains and escape mutants (if any) of MERS-CoV.

Taken together, these results suggest to us that the MERS-CoV RBD protein-specific m336 mAb is an excellent candidate for passive immunotherapy to provide immediate and effective protection to individuals who may be exposed to MERS-CoV and to treat patients who have been exposed. Testing in humans is needed for its potential use as a therapeutic for the treatment of MERS-CoV-infected patients.

## Methods

### Monoclonal antibody production

For expression of m336 IgG1, the previously described m336 IgG1 vector^23^ was used to infect CHO-K1 cells (ATCC, Manassas, VA) with PolyFect transfection reagent (Qiagen, Valencia, CA). After screening of 960 clones for antibody productivity by ELISA and subsequent characterization, a stable cell line was generated and inoculated into a Bioflo 410 bioreactor (New Brunswick Scientific, NJ) for large-scale production of m336 IgG1. Purification was carried out by using a protein G column (GE Healthcare), and endotoxin was removed by Detoxi-Gel Endotoxin Removing Columns (Thermo Scientific) according to the manufacturer’s instructions.

### Mice, virus, and cells

Transgenic mice expressing human DPP4 established by us were used throughout the study. Animals were housed in on-site animal facilities at Galveston National Laboratory under a 12:12 light/dark cycle with room temperature and humidity kept between 21–25 °C and 31–47%, respectively, and with ad libitum access to food and water. All experiments were performed in accordance with the Guide of NIH and AAALAC and were approved by the Institutional Animal Care and Use Committee at the University of Texas Medical Branch, as described previously^34^. Briefly, groups of 6–8-weeks Tg mice were challenged intranasally (in) with 10^4^ TCID_50_/ml (~1,000 LD_50_) of MERS-CoV-EMC/2012, originally provided by Heinz Feldmann (NIH, NIAID Rocky Mountain Laboratories, Hamilton, MT) and Ron A. Fouchier (Erasmus Medical Center, Rotterdam, Netherlands). The titers of individual virus stocks, stored at −80 °C, were determined by using Vero E6-based infectivity assays and expressed as 50% tissue culture infectious doses (TCID_50_)/ml.

### Viral infections and isolation

All of the animal studies involving infectious MERS-CoV were conducted within approved animal biosafety level 3 (ABSL-3) at the Galveston National Laboratory. Experimental designs and strategies in different Tg mouse groups involving intranasal challenge with live MERS-CoV were described in individual experiments in the Results section. For live virus isolation, lung tissues were collected at day 2 post MERS-CoV challenge, weighed, and homogenized in phosphate-buffered saline (PBS) containing 10% fetal calf serum (FCS) by using TissueLyser (Qiagen, Retsch, Haan, Germany), as previously described^34^. The resulting suspensions of infected tissues were tittered in the standard Vero E6 cell-based infectivity assays to quantify yields of infectious virus expressed as log_10_ TCID_50_ per gram (g) of tissue.

### RNA extraction and viral titers determination by real-time Q-PCR

Lung tissue samples from each group of mice were transferred to individual vials having RNA later solution (Qiagen) and subsequently homogenized and subjected to total RNA isolation, by using TRIzol Reagent (Life Technologies), to assess MERS-CoV-specific genome targeting of virus-specific upstream E gene (upE) and endogenous control gene (mouse β-Actin) by using a one-step RT-PCR kit (Invitrogen), as previously described^34^. Ct values for each sample were analyzed against Ct values generated in our lab from the standard curve of MERS-CoV mRNA copy number. Relative MERS-CoV upE mRNA expression value was calculated for each replicate and expressed as the equivalent of log10 TCID_50_ per gram (g) of the tissue by the standard threshold cycle (∆∆CT) method. Ct value analysis was done by using Bio-Rad CFX Manager 3.0 software.

### Histopathology

Mice were necropsied, lung tissues were inflated and fixed in 10% neutral buffered formalin for 3 days before paraffin-embedded and processed for routine hematoxylin and eosin stain (H&E) for assessing the histopathology^46^.

## Additional Information

**How to cite this article**: Agrawal, A. S. *et al*. Passive Transfer of A Germline-like Neutralizing Human Monoclonal Antibody Protects Transgenic Mice Against Lethal Middle East Respiratory Syndrome Coronavirus Infection. *Sci. Rep*. **6**, 31629; doi: 10.1038/srep31629 (2016).
